# The Name “Allopath”

**Published:** 1879-07

**Authors:** C. W. Wooldidge

**Affiliations:** Whitehall, Mich.


In a letter of Dr. C. W. Wooldidge, in the Michigan *Medical News* the following remarks upon our "pet name" are given :

The Name "Allopath."—In the last number of the *News* you ask for suggestions looking to the relief of the profession from the term "allopathy." A homœopathic text book (Grauvogle's) has recently come to my notice, in which the author uses the term "physiological school" to designate the regular school of medicine, as distinguished from his own, and the term has seemed to me so truly descriptive, and so wholly unobjectionable, that I wish the profession would unite in adopting it. The word is a long one, it is true, but it has the merit of carrying a meaning, which the masses can in some degree understand, and which so far as it is understood, will be complimentary to the profession. It will be easier to adopt it, too, because it carries with it no implied reproach to others, as the word "rational" would, if the regular profession should assume to take it to themselves as distinguishing them from all others.

The patent right on common property has already become a fixture, in the appropriation of the word "eclectic" by a sect in medicine whose ecclecticism is far less thorough and universal than our own. If it were not for the fact that it has been thus appropriated, or if the sectarian school could be dispossessed of it, eclectic would be a good word for our use, but as it is, "physiological" seems to be the best term that I have yet seen, and the fact that some of the homœopaths are helping us to it is worth something. They have succeeded in dubbing us with the term allopath, and if now each and all of the medical profession would unite in resenting that misnomer, as applied to himself and would agree upon some intelligible substitute, perhaps it would not be so very difficult to get rid of the nickname.

It is possible that you may be flooded with suggestions on this subject between now and the next issue, but if you can find room for this letter in the *News* I wish you would print it.

I am, very respectfully,

C. W. WOOLDIDGE, M.D.

*Whitehall, Mich., June* 22, 1879.

